# Case Report: Rechallenge With BRAF and MEK Inhibitors in Metastatic Melanoma: A Further Therapeutic Option in Salvage Setting?

**DOI:** 10.3389/fonc.2021.645008

**Published:** 2021-05-31

**Authors:** Anna Stagno, Sabrina Vari, Alessio Annovazzi, Vincenzo Anelli, Michelangelo Russillo, Francesco Cognetti, Virginia Ferraresi

**Affiliations:** ^1^ Department of Medical Oncology 1, IRCCS-Regina Elena National Cancer Institute, Rome, Italy; ^2^ Nuclear Medicine Unit, IRCCS-Regina Elena National Cancer Institute, Rome, Italy; ^3^ Radiology and Diagnostic Imaging Unit, IRCCS-Regina Elena National Cancer Institute, Rome, Italy

**Keywords:** metastatic melanoma, *BRAF* V600 mutation, drug resistance, targeted therapy, BRAF inhibitor, MEK inhibitor, rechallenge

## Abstract

**Background:**

The combination of BRAF and MEK inhibitors represents the standard of care treatment for patients with metastatic *BRAF*-mutated melanoma, notwithstanding the high frequency of emergent resistance. Moreover, therapeutic options outside clinical trials are scarce when patients have progressed after both targeted therapy and therapy with immune checkpoint inhibitors. In this article, we report our experience with targeted therapy rechallenging with BRAF and MEK inhibitors in patients with metastatic *BRAF*-mutated melanoma after progression with kinase inhibitors and immunotherapy.

**Methods:**

Four patients with metastatic *BRAF*-mutated melanoma were rechallenged with BRAF and MEK inhibitors after progression with targeted therapy and subsequent immunotherapy (checkpoint inhibitors).

**Results:**

Two patients (one of them was heavily pretreated) had partial response over 36 months (with local treatment on oligoprogression disease) and 10 months, respectively. A third patient with multisite visceral disease and high serum levels of lactate dehydrogenase had a short-lived clinical benefit rapidly followed by massive progression of disease (early progressor). The fourth patient, currently on treatment with BRAF/MEK inhibitors, is showing a clinical benefit and radiological stable disease over 3 months of therapy. Adverse events were manageable, similar to those reported during the first targeted therapy; the treatment was better tolerated at rechallenge compared with the first treatment by two out of four patients.

## Introduction

*BRAF* V600E or V600K mutation is a predictor of response to therapy with BRAF inhibitors (BRAFi). Several mutations of the *BRAF* gene (over 90% are V600E) are responsible for the constitutive activation of the MAPK/ERK signaling pathway, which leads to uncontrolled cell proliferation and survival. *BRAF* mutations have been reported in 40–50% of patients with cutaneous melanomas (1–3).

In patients with unresectable or metastatic *BRAF*-mutated (V600E or V600K) melanoma, the combination of BRAFi and MEK inhibitors (MEKi) is the current standard of care in the first-line setting, with three approved combinations: dabrafenib and trametinib, vemurafenib and cobimetinib, and encorafenib and binimetinib. Overall, this therapy obtained response rates of 68% to 76%, median progression-free survival (PFS) of 11–15 months and a 3-year overall survival (OS) rate of approximately 40% (4–8). More recent data demonstrated that the first-line combined targeted therapy with dabrafenib and trametinib was associated with long-term PFS of 19% and an OS rate of 34% at 5 years in the overall population (4). In addition, this combined treatment has a fast onset of efficacy, but half of the patients experience progression of disease within approximately 1 year of treatment due to primary or acquired resistance (9). Frequent mechanisms of resistance are reactivation of the MAPK pathway for upstream and downstream mutation of *BRAF* or of the PI3K–PTEN–AKT pathway. Several mechanisms of resistance can be found simultaneously in the same patient (intrapatient heterogeneity) or even in the same lesion (intratumor heterogeneity) (10–12).

Anti-PD-1 (nivolumab and pembrolizumab) and anti-CTLA-4 (ipilimumab) antibodies represent effective approved lines of treatment for patients with *BRAF* mutations who are pre-treated with BRAFi/MEKi (13). Patients progressing after treatment with BRAFi/MEKi and failure of immunotherapy have a poor prognosis, as palliative chemotherapy is the only additional option currently available outside of clinical trials.

Rechallenge with the combination of BRAFi and MEKi after a previous treatment with the same class of inhibitors has been associated with limited activity; retrospective analyses and one prospective trial obtained re-induction of response in over 30% of patients and a median PFS of approximately 5 months (14–16). In this article, we describe four cases of patients with metastatic melanoma patients rechallenged with BRAFi and MEKi (dabrafenib and trametinib) after a previous line of treatment with BRAFi ± MEKi. We present and discuss experience with treatment beyond progression even in the setting of advanced heavily pretreated disease. Finally, our clinical data are compared with available evidence as obtained through a literature review on BRAFi/MEKi rechallenge in advanced melanoma.

## Methods

We analyzed four patients who were treated with dabrafenib and trametinib rechallenge between December 2017 and December 2020; they fulfilled the following eligibility criteria: metastatic melanoma with *BRAF* V600 mutation tested in primary tumor or on distant metastases; previous treatment with a BRAFi with or without a MEKi with progression after an initial stable, partial, or complete response; progression of disease after at least another therapy regimen, including treatment with an anti-PD-1 antibody (nivolumab or pembrolizumab) ± ipilimumab before rechallenge; Eastern Cooperative Oncology Group [ECOG] performance status (PS) <2; no previous severe toxicities from dabrafenib ± trametinib precluding treatment administration; and no availability of investigational clinical trials to be offered to the patient.

Clinical data and treatment outcomes were extracted from electronic and paper clinical records; patients underwent periodic clinical examinations, blood tests and imaging examinations with computed tomography or ^18^F-FDG-PET and brain MRI to assess the objective response, according to the clinical practice (every 3 months or earlier in case of clinical signs of suspected progression of disease).

Rechallenge with BRAFi/MEKi is off-label: approval of treatment was obtained from our internal Pharmacy and Health Direction and all patients provided written informed consent. Due to the retrospective nature of the analyses, an informed consent for clinical data collection was not requested.

## Case Report

### Patient 1

In November 2007, a 66-year-old man underwent excision of cutaneous melanoma on the first toe of his right foot (Breslow thickness >4 mm, ulcerated, Clark level V) with positive inguinal sentinel node and left femoral–iliac lymphadenectomy negative for metastatic lymph nodes (stage IIIA, AJCC 7th edition). Due to the occurrence of metastatic disease on the right maxillary sinus, from October 2008 to January 2009, he received first-line chemotherapy with cisplatin, vinblastine and dacarbazine (CVD regimen) and radiotherapy (total dose administered was 30 Gy in 7 fractions), with a good local control of disease. In May 2009 and August 2012, the patient underwent surgical resection of isolated subcutaneous metastases on the back and axillary region, respectively. From July to September 2014, due to the spread of subcutaneous metastases to the upper and lower limbs, the patient was treated with ipilimumab; he obtained a stable disease as best response. This response was maintained until July 2015 when a subcutaneous, soft tissue, bone and pulmonary progression of disease occurred. In August 2015, due to the presence of *BRAF* mutation, treatment with the BRAFi dabrafenib (150 mg twice daily) and the MEKi trametinib (2 mg once daily) was started. Treatment was well tolerated except for recurrent episodes of grade 2 hyperpyrexia (with body temperature as high as 40.0°C) not resolved by temporary dabrafenib ± trametinib discontinuation and finally leading to progressive dose reductions (lower dose schedule: dabrafenib 50 mg twice daily and trametinib 1 mg once daily). At the first radiological examination with 18F-FDG-PET, a partial response in the lungs, soft tissues and right maxillary sinus was documented with stable disease in the bone. The response lasted 19 months until March 2017, when the treatment was discontinued due to progression of the right maxillary sinus lesion and of subcutaneous lower limb metastases. From March to July 2017, the patient received six infusions of the anti-PD-1 antibody (pembrolizumab), followed by systemic progression of disease on all metastatic sites and the appearance of a single brain lesion, for which he received stereotactic radiation. Between September and November 2017, the patient was treated with a single agent, dacarbazine, with progression of disease on the right maxillary sinus and with the appearance of a new brain lesion in the temporal lobe. As further new treatment options were not available, and clinical conditions were good (ECOG PS 0–1), in December 2017, the patient resumed treatment with dabrafenib and trametinib and received stereotactic radiation in a single fraction on the new brain lesion. LDH value at the time of the start of treatment was high [>1 to <2 × upper normal limit (UNL)]. Due to previous severe dose-limiting hyperpyrexia in concomitance with the same agents, the dose of dabrafenib was *ab initio* reduced to 75 mg twice daily while maintaining the full dose of trametinib (2 mg once daily). The first radiological evaluation with computed tomography (CT) scan in May 2018 showed a partial response in the brain, lungs and right maxillary sinus lesions and a stable disease for bone metastases. In September 2020, due to ulceration of the maxillary lesion suggestive of local progression of disease, we decided to treat the patient locally with electrochemotherapy for cytoreductive purposes and local control. After 34 months of rechallenge, at the time of this report, treatment with dabrafenib and trametinib was being continued: at the last radiological evaluation with a CT scan in November 2020, partial response in all disease sites, including the maxillary lesion was maintained. Treatment was tolerated; some episodes of pyrexia were controlled by temporary discontinuation of the BRAFi alone and without a further dose-reduction of both dabrafenib and trametinib as for previous treatment with the same agents.

### Patient 2

In November 2008, a 35-year-old man was diagnosed with melanoma on the left cervical region (Breslow thickness 1.2 mm, not ulcerated, negative neck sentinel node; pT2a, positive for the *BRAF* V600E mutation). The published data on rechallenge of targeted therapy are summarized in **Table 1**. In December 2014, the patient underwent left cervical lymphadenectomy with 1/19 metastatic lymph nodes (stage IIIA, AJCC 7th edition) for cytologically confirmed neck lymph node metastases. From January to September 2016, due to clinical and radiological evidence of new multiple cutaneous metastasis, the patient (at that time ECOG PS 0) was treated with the BRAFi dabrafenib (150 mg twice daily) and MEKi trametinib (2 mg once daily), obtaining stable disease/minor response as best response. The treatment was well tolerated without dose reductions and only grade 1 dermatological toxicity and grade 1 pyrexia treated with temporary dabrafenib interruption as per the toxicity algorithm. Despite clinical control of known cutaneous metastases, in September 2016, multiple secondary brain lesions were detected by CT scan. In the absence of central neurological symptoms, the patient was treated with nivolumab until July 2017 when the MRI and CT scan showed a progression of brain lesions, subcutaneous metastases were controlled. The patient underwent whole-brain radiotherapy (total dose 30 Gy in 10 fractions) and continued nivolumab until January 2018 when the therapy was interrupted due to a further progression of disease in the brain, cutaneous, subcutaneous, soft tissue (on the right pre-hyoid region) and laryngeal as confirmed by 18F-FDG-PET/CT in January 2018. LDH value was >1 to <2 UNL. In our opinion, the fast-aggressive disease was not amenable for crossover to ipilimumab. Hence, in February 2018, clinical condition was good (ECOG PS 1), we decided to offer the patient a rechallenge with full-dose dabrafenib and trametinib. The first radiological control showed stable disease on brain lesions (as documented by MRI at 4 months since the beginning of treatment) and a metabolic complete response on all other sites of disease, as shown by 18F-FDG-PET/CT in June 2018, with clinical improvement of dysphonia. Clinical evaluation showed a partial response of cutaneous metastases. Tolerability to rechallenge was good without the appearance of pyrexia and only grade 1 erythema at the site of a tattoo. In October 2018, further progression of disease in the brain occurred, and a second course of palliative brain radiotherapy (total dose 20 Gy) was administered. In January 2019, the patient died due to the progression of metastatic lesions in the brain.

**Table 1:** Summary of published data on the rechallenge of targeted therapy in melanoma.

	Valpione et al. (14)	Schreuer et al. (15)	Tietze et al. (17)	Cybulska-Stopa et al. (16)
Analysis	Retrospective	Prospective	Retrospective	Retrospective
Simple Size (n)	116	25	60	51
TT1	58.6% BRAFi mono 35.3% BRAFi + MEKi 4.3% BRAFi + IT 1.8% missing data	36% BRAFi mono 64% BRAFi + MEKi	68% BRAFi mono 32% BRAFi + MEK	35% BRAFi mono 65% BRAFi+MEKi
Median PFS (months)	NA (9.4 months median duration of TT)	6.9	9.9	10.5
Treatment during interval	71.5% IT	56% anti PD-1 28% anti-CTLA-4	27% anti-PD-1 22% anti-CTLA-4 7% anti-PD-1 + anti CTLA-4 8% CHT + IT 8% RT or surgery	88% anti-PD-1 12% anti-CTLA-4 Subsequent line: * 16% CHT * 2% anti-PD-1
Duration of interval (median months)	7.7	6.1	3.4	8.6
TT2	BRAFi mono BRAFi + MEKi BRAFi + MEKi + ribociclib	100% BRAFi + MEKi	32% BRAFi mono 68% BRAFi + MEKi	4% BRAFi mono 96% BRAFi + MEKi
Median PFS (months)	5	4.9	5	5.9
Median OS (months)	9.8	Not reached	15.5 (initial BRAFi mono–BRAFi in rechallenge) 10.6 (initial BRAFi mono–BRAFi + MEKi at rechallenge) 5.2 (initial BRAFi + MEKi–BRAFi + MEKi at rechallenge)	9.3

TT, targeted therapy; BRAFi, BRAF inhibitor; MEKi, MEK inhibitor; IT, immunotherapy; anti PD-1, anti-programmed cell death protein 1; anti CTLA-4, anti-cytotoxic T lymphocyte- associated protein 4; CHT, chemotherapy; RT, radiotherapy; PFS, progression-free survival; N.A., not available; OS, overall survival.

### Patient 3

In January 2011, a 57-year-old man went through radical excision of a lesion in the scalp and left cervical lymphadenectomy for palpable nodes with a final diagnosis of superficial spreading melanoma (Breslow thickness 0.8 mm, not ulcerated, Clark level IV) with 7/38 metastatic lymph nodes, stage pT1 pN3 M0, IIIC as per AJCC 7°edition. In November 2011, a 18F-FDG-PET/CT showed multiple nodal and soft tissue lesions and the *BRAF* mutation test revealed the presence of *BRAF* V600 mutation. For this reason, from January 2012 to January 2020, the patient was treated with vemurafenib (960 mg twice daily) in the MO25515 trial with a complete response. In January 2020, the CT scan showed a massive progression of disease with the occurrence of brain, lung, liver and soft tissue metastases and concomitant high LDH values. Therefore, we decided to treat the single temporal metastases with stereotactic radiotherapy, and, in March 2020, the patient started immunotherapy with nivolumab. In May 2020, a progression of disease in the lungs, soft tissue and liver was documented with hepatic failure and obstructive jaundice (bilirubin 8 mg/dl) with dilatation of biliary system. The LDH value was superior to two-times UNL (2028 U/L). After biliary stent placement and subsequent normalization of liver function analyses, taking into account the prior very long-term response (8 years) to BRAFi monotherapy and the poor therapeutic options in this patient with rapid clinical progression disease but still acceptable ECOG PS 1, we decided for a rechallenge with BRAFi and MEKi (dabrafenib and trametinib). The patient received the treatment only for 1 month with good tolerability, which was a short-lived clinical benefit and an initial reduction of LDH values but, unfortunately, the patient died in July 2020 for rapid progression of disease.

### Patient 4

In June 2012, a 46-year-old man underwent primary excision of melanoma on the back (Breslow thickness >4 mm, ulcerated) and right axillary lymphadenectomy with 14/14 positive metastatic lymph nodes (stage IIIC, AJCC 7th edition). In January 2013, for cytologically confirmed lymph node relapse of disease, the patient went through a left maxillectomy and right laterocervical lymphadenectomy with 4/34 positive lymph nodes for metastatic disease (*BRAF* V600E mutation positive). In June 2013, due to local recurrent disease on the left maxillary sinus and the appearance of new lung metastasis, the patient, who was in good clinical condition (ECOG PS 0), was enrolled in the coBRIM clinical trial, receiving the BRAFi vemurafenib (960 mg twice daily) until July 2014; he obtained partial response as best response. From August to November 2014, after the evidence of progression of disease in the lungs, the patient received immunotherapy with four cycles of ipilimumab, obtaining a partial response that lasted until July 2015 when a CT scan showed a further progression of disease in the lungs. For this reason, the patient was enrolled in the CheckMate 067 randomized clinical trial and received nivolumab as single agent with a complete response on lung metastases until December 2016, when the treatment was discontinued due to the appearance of isolated small intestine metastases. Therefore, the patient underwent bowel resection (large and small intestine) with histological confirmation of melanoma metastases. The clinical–radiological follow-up was negative until November 2018, when a new pathological abdominal lymphadenopathy (paraaortic, retroduodenal, external iliac) appeared and retreatment with nivolumab was offered to the patient. Anti-PD-1 antibody was administered until April 2020 when a new isolated rectus abdominis muscle lesion was documented on 18F-FDG-PET/CT. Hence, the rectus abdominis and left oblique muscle lesions were surgically removed with a histological result compatible with melanoma metastasis. In August 2020, due to radiological evidence of abdominal soft tissue and nodal progression of disease and considering the good clinical condition (ECOG PS 1), the patient started targeted therapy rechallenge with dabrafenib and trametinib. LDH values at that time, were within normal limit. At the beginning of therapy, some occasional episodes of nausea and vomiting (G2) treated with metoclopramide were reported, with good symptom control. At the first clinical re-evaluation after 4 weeks of treatment, the patient showed a clinical response with reduction in size of inguinal lymphadenopathy. In December 2020, the first radiological revaluation with CT scan documented stable disease. The treatment is still ongoing and well tolerated.

## Discussion

We reported four cases of pretreated patients with *BRAF*-mutated advanced melanoma, who were rechallenged with off-label targeted therapy after immunotherapy with single agents (at least anti-PD-1 antibodies), as effective approved therapeutic options were not available.

Our four patients were heterogeneous with respect to duration and type of first course of BRAFi/MEKi (BRAFi alone versus combination with MEKi), and clinical outcome after rechallenge treatment (**Table 2**). Published evidence suggests that ≥3 metastatic sites and elevated LDH levels are prognostic unfavorable factors associated with poor PFS and OS, both at the first treatment and at rechallenge with targeted therapy (18). In our experience, two patients (patients 1 and 2) had partial response over 36 months (including treatment of limited progression) and PFS of 10 months after rechallenge. They had poor prognostic factors at baseline, such as high LDH level (>1 to <2 × UNL) and more than three metastatic sites (including brain metastases), but a satisfactory PS. In particular, patient 1 obtained partial response on the lung and right maxillary sinus lesions and maintained stability on bone disease without the appearance of new brain lesions for over 3 years. In patient 2, we observed metabolic complete response on lesions of the larynx (with improvement of dysphonia), and subcutaneous and soft tissues, and clinical partial response on multiple cutaneous metastases without new brain lesions for approximately 1 year. Patient 3 had bad prognostic factors (such as bulky disease with unfavourable extra-cerebral sites including the liver, and very high LDH values), and obtained a very short-lived clinical benefit.

**Table 2:** Summary of clinical cases.

Patient	Gender/age (years)	TT1	Disease sites at	Best response	DOR (months)	Toxicity (grade)	Further lines of therapies before TT2	Time to TT2 (months)	PS at TT2	LDH (ULN) at TT2	Disease sites at TT2	Best response TT2	DOR TT2 (months)	Toxicity TT2 (grade)	OS (months)
1	M/76	BRAFi (D) MEKi (T)	Bone, lung, subcutaneous lesions, soft tissue, maxillary sinus	PR	19	Fever G2	Pembro Dacarbazine	9	0–1	High (>1 <2 ULN)	Bone, lung, subcutaneous, soft tissue, maxillary sinus, brain	PR	36 TBO	Fever G1	36
2	M/45	BRAFi (D) MEKi (T)	Cutaneous metastasis	SD	9	Skin G1, fever G2	Nivo	17	1	High (>1 <2 ULN)	Brain, Hyoid region, laryngeal, cutaneous and subcutaneous	PR	10	Skin G1	10
3	M/66	BRAFi alone (V)	Soft tissues, lymph nodes	CR	96	–	Nivo	6	1	High (>2 ULN)	Brain, lung, liver, soft tissues	PD	1	–	1
4	M/54	BRAFi alone (V)	Maxillary sinus, lung	PR	12	–	Ipi Nivo	72	1	Normal	Soft tissues, lymph nodes	SD	3 ongoing	Nausea, vomiting G2	3

PS, performance status; TT1, first targeted therapy; TT2, second targeted therapy (rechallenge); BRAFi, BRAF inhibitor; MEKi, MEK inhibitor; D, dabrafenib; T, trametinib; V, vemurafenib; Pembro, Pembrolizumab; Nivo, Nivolumab; Ipi, Ipilimumab; M, male; ULN, upper limit of normal; TBO, treatment beyond oligoprogression; DOR, Duration of response; OS, overall survival.

In the literature, the presence of brain metastasis is generally associated with a limited efficacy of therapy and poor prognosis (19). In our experience, we cannot rule out the possible role of dabrafenib and trametinib in preventing or slowing down the occurrence of new brain lesions. Patients 1 and 2 had brain metastases before rechallenge; they started radiotherapy (stereotactic radiosurgery and whole-brain radiation therapy) before rechallenge, and continued it for a long period of time (34 months for patient 1 and about 12 months for patient 2) without the occurrence of new brain lesions.

The efficacy of rechallenging with targeted therapy has been evaluated in a retrospective study on 116 metastatic melanoma patients retreated with BRAFi (with or without MEKi) (14). The median OS after rechallenge treatment was 9.8 months, with median PFS of 5 months, and a response rate of 43%. The highest benefit was found for patients who progressed after an initial response to targeted therapy (BRAFi with or without MEKi) and who did not respond to immunotherapy as second-line treatment. The worst prognosis was associated with patients retreated with the single-agent BRAFi, who had many metastatic organs (three or more) and a high value of LDH. The duration of the drug holiday between the first targeted therapy and rechallenge was apparently not associated with incidence of response.

Another recent multicenter retrospective analysis investigated the retreatment with BRAFi and MEKi combination in 51 patients with metastatic *BRAF*-mutated melanoma, previously progressed after receiving kinase inhibitors (BRAFi and MEKi or BRAFi alone) and immunotherapy (anti-PD-1 or anti-CTLA-4) (16). The median PFS and OS for the rechallenge combination treatment was 5.9 and 9.3 months, respectively. The overall response rate was higher for the first treatment of BRAFi and MEKi (72%) and lower at rechallenge (27%). The authors concluded that good general conditions (ECOG PS 0 or 1), female sex, normal LDH level and the absence of brain metastases were associated with higher chance to control the disease after rechallenge. Moreover, a longer time from end of the first treatment to rechallenge seemed to be associated with a higher rate of overall response rate but not to be a predictor of better PFS or OS.

In 2017, Schreuer et al. reported the results of a prospective phase II clinical trial, exploring the activity of BRAFi/MEKi combination rechallenge in 25 *BRAF* V600-mutated patients who progressed after a previous first-line treatment with a BRAFi-containing treatment (15). All patients received immunotherapy (anti-CTLA-4 or anti-PD-1 antibodies) as second-line treatment. Eight patients (32%) achieved an objective partial response (six of the eight patients had brain metastases) and, overall, 72% of patients achieved disease control. Responding patients showed a median PFS of 4.9 months with rapid clinical improvement. A short time to start of treatment (≤3 months) and high value of LDH were associated to a worse outcome.

As previously stated, the response to targeted therapy is often limited by the development of resistance through different molecular mechanisms. Resistance is primary/pre-existing or intrinsic when progressive disease is the best response, and secondary/acquired when progressive disease is observed after a clinical benefit (20). Approximately 20% of patients with *BRAF* V600E mutation melanoma had intrinsic resistance to BRAF inhibition (21). Several mechanisms of intrinsic resistance have been described among whom loss of PTEN, cyclin D1 amplification, loss of NF1, *RAC1* P29S mutation, HGF secretion, MAP3K8 overexpression, loss of the USP28–FBW7 complex, low levels of CD8 tumor-infiltrating T cells and increased expression of the immune inhibitory molecule PD-L1 (21–31). On the other hand, mechanisms of adaptive resistance that develop during early exposition to targeted therapy and limit their efficacy are also described (18). In the last few years, several mechanisms of resistance to BRAFi have been described, such as signals emanating from the host microenvironment, cancer stem cells and microRNA (32).

The reactivation of the MAPK pathway is the most frequent mechanism of acquired resistance (31, 32). This can occur for mutation upstream (through upregulation of receptor tyrosine kinase or mutation in RAS) and downstream (through activating mutation in MEK1 or MEK2) of BRAF or the PI3K–PTEN–AKT pathway and at the level of BRAF within the pathway (i.e., through copy number amplification of the mutant *BRAF* allele) (12, 33). Moreover, melanomas can present a high grade of both intratumor and intrapatient molecular heterogeneity (22). Preclinical models suggested that clones resistant to BRAFi could have a fitness disadvantage compared to those sensitive to BRAFi. The selective growth advantage in the face of BRAFi therapy could be lost on discontinuation of the BRAFi (34). These findings could also have an impact on the evaluation of intermittent treatment regimens in melanoma, possibly as it occurs in other cancers including renal cell carcinoma (34). Other models for transient acquired resistance include signalling plasticity (35–37), phenotype switching (38), quiescence (39, 40) or epigenetic changes (41). It has been demonstrated that such mechanisms of resistance in patients who are candidates for rechallenge are reversible, hence, after a ‘drug holiday’ some patients may regain sensitivity (35).

Fallahi-Sichani et al. showed that tumor cells may have a heterogeneous response to BRAFi/MEKi: some cells die, some arrest and some can adapt to drugs through dedifferentiation with a slow growth (42). This last phenotype is reversible in a ‘drug-free’ environment (43).

Although reversible resistance was demonstrated by preclinical studies, recent clinical experiences do not support the strategy of intermittent doses of BRAFi/MEKi. In 2013, a preclinical experience with xenograft models showed that continued exposure to BRAFi fuels treatment-resistant tumor cells, while an intermittent schedule delays the onset of resistance preserving tumor control (34). The randomized phase II trial SWOG S1320 conducted by Algazi et al. compared intermittent dosing of dabrafenib and trametinib (3 weeks off/5 weeks on) versus the standard continuous treatment in patients with metastatic *BRAF*-mutated melanoma, in order to verify the role of drug “holiday” in overcoming acquired resistance and improving the outcome (44). All patients received an 8-week lead-in course of continuous dosing after which patients who achieved an objective response or stable disease were randomized to continuous (n=105) or intermittent (n=101) dabrafenib plus trametinib. The standard continuous therapy proved to be associated with a longer PFS (9 months for continuous dosing versus 5.5 months for intermittent therapy; hazard ratio, 1.36; 80% CI: 1.10–1.66; p=0.063). The PFS advantage in favor of continuous dosing was observed independently from age, sex, LDH value and prior immune checkpoint inhibitor therapy. Differences in drug metabolism and in mechanisms of resistance between humans and mice were proposed as possible explanations (45).

In the COMBI-MB trial, evaluating the clinical activity of the combination of dabrafenib and trametinib on brain metastases in *BRAF*-mutated melanoma patients, an intracranial response rate of 56% and a PFS at 6 and 12 months of 71 and 47%, respectively, was demonstrated in cohort B of asymptomatic patients with prior local therapy (46). Tietze et al. showed a correlation between the responses to the first and second treatment observing that the higher the response after the first BRAFi, the higher the possibility to respond to the rechallenge (17). In addition, the duration of response was longer in the rechallenge setting than after the first treatment. The correlation between duration of treatment break and clinical outcome is unclear. Valpione et al. reported that the duration of treatment break (6.7–8.8 months) seems to be correlated with clinical outcome (14) while retrospective analyses and one prospective clinical trial did not confirm this finding (15–17). In our patients, the duration of the interval between treatments was more than 6 months for all cases but we observed mixed outcomes: two patients had long-term control of the disease (patient 1 and 2) and one patient (patient 3) had only a short-lived stabilization of symptoms (1 month) rapidly followed by a multi-site progression of disease. We observed a stable response at first radiological evaluation in patient 4, but the follow-up was too short to address this issue, at the time of this report. Cybulska-Stopa et al. reported adverse events in a greater number of patients during the first treatment with BRAFi with or without MEKi compared with rechallenge, demonstrating a lower toxicity, good tolerance of rechallenge and lack of toxicity accumulation of the treatments (16). The most frequent adverse events were mainly G1–2 (dermatological toxicity, pyrexia, diarrhea, increased levels of liver function enzymes), while the few G3 adverse events included rash, hepatotoxicity and skin cancer. Our experience seems to confirm this latter evidence: similar adverse events have been reported during the first and rechallenge treatment with better tolerability at rechallenge for patients 1 and 3. As illustrated, most of the published experiences of rechallenge with BRAFi ± MEKi were in patients who experienced disease progression during a previous treatment with the same class of agents. The correlation between previous treatment-limiting toxicity leading to discontinuation and chance of subsequent response remains unclear. The study by Valpione et al. included patients who discontinued the first course of targeted therapy due to different reasons (toxicity: 16 patients; treatment break after complete response: nine patients; disease progression: 83 patients; other reasons: five patients) (14). The overall response rate at rechallenge was 43% (49/113) in the evaluable patients, while it was 37.3% (31/83) for patients who had discontinued BRAFi due to disease progression, and 60% (18/30) in those who had discontinued the first treatment due to reasons other than progression. This latter frequency matches the expected overall response rate to the combination of BRAFi and MEKi in *BRAF*-mutated treatment-naïve patients, No correlative data between specific reasons of interruption (toxicity, complete response or other causes) and response was, however, provided. In small retrospective series of patients in complete response during BRAFi ± MEKi, treatment-related toxicity was the most reported cause of interruption (47–49). Approximately 50% of these patients experienced disease progression at various times from discontinuation and retreatment with BRAFi/MEKi, and no particular correlation between the occurrence of previous toxicity and outcome was reported.

In our clinical practice, treatment with BRAFi/MEKi is administered until disease progression, and rechallenge is offered only to patients who had a previous progression of disease with the same class of agents. We had no experiences of rechallenge in patients who interrupted the treatment due to toxicity.

In retrospective analyses, targeted therapy demonstrated clinical benefit when used beyond limited progression because of loco-regional treatment (50, 51). Intratumoral and intertumoral heterogeneity can explain the prolonged antiproliferative activity of BRAF inhibition beyond disease progression. Moreover, different studies suggest that patients who receive BRAFi may progress in some sites of metastases: the use of loco-regional treatment is important in these cases, while the BRAFi is active for the remaining drug-sensitive disease (52, 53). The same benefit seems to occur also in the setting of rechallenge. In patient 1, the loco-regional treatment of limited progression on maxillary sinus with electrochemotherapy maintained ongoing long-term response to BRAFi/MEKi for drug-sensitive lesions. An interesting aspect of Schreuer’s study, which should be deeply analyzed, is the use of liquid biopsy as a type of biomarker capable to predict PFS in the rechallenge treatment (15). In this study, the liquid biopsies have been collected at rechallenge baseline, after 2 weeks, and every 2 months until disease progression. The study shows that the persistent detection of elevated copy number *BRAF* V600-mutated circulating tumor DNA (ctDNA) after 2 weeks of therapy correlates with a worse prognosis. There is a correlation between a high copy number of ctDNA with high proliferative activity/status of tumor cells and a high tumor burden. Several studies in metastatic melanoma patients, showed that ctDNA is a diagnostic, predictive and prognostic biomarker, which is useful to detect eligibility to rechallenge (15, 54–58). Preclinical and clinical evidence suggests that the combination of BRAFi/MEKi and checkpoint inhibitors (anti-PD-1 and anti-PD-L1) could be a good strategy to overcome resistance to BRAF inhibition, providing durable responses in a high rate of metastatic melanoma patients thanks to the activity of BRAFi to enhance immune activation. Nevertheless, the role of triplets remains to be defined and is probably confined to patients with poor prognosis (high LDH, metastatic sites ≥3, stage [M1c vs other], multiple brain metastases and progression of disease during adjuvant treatment) (59–62).

In conclusion, patients with metastatic *BRAF*-mutated melanoma who progressed on kinase inhibitors and immunotherapy represent a clinical setting with very limited therapeutic options. Retreatment with BRAFi and MEKi may be considered in selected patients with adequate performance status. Current evidence suggests that this therapeutic choice may prolong control of disease without unexpected toxicities. Future research on the mechanisms of resistance to BRAFi could help identify optimal candidates for rechallenge therapy.

## Data Availability Statement

The raw data supporting the conclusions of this article will be made available by the authors, without undue reservation.

## Ethics Statement

Ethical review and approval was not required for the study on human participants in accordance with the local legislation and institutional requirements. The patients/participants provided their written informed consent to participate in this study.

## Author Contributions

AS, SV, AA, VA, MR, FC, and VF contributed to conception, writing, and review of the article. ****All authors contributed to the article and approved the submitted version.

## Conflict of Interest

The authors declare that the research was conducted in the absence of any commercial or financial relationships that could be construed as a potential conflict of interest.
